# Recognition

**Published:** 1884-02

**Authors:** Ad. Lippe

**Affiliations:** Philadelphia


### RECOGNITION.

AD. LIPPE, M. D., PHILADELPHIA.

Recognition: The Old Code *versus* the New Code is *the* burning question of the day, and it disturbs the peace of the "regulars," as they are pleased to term themselves. The medical societies are sadly troubled by it. What does it all amount to? Before progressing any further to examine the merits of the New Code it would be well to reflect for a moment. There seems to pervade the minds of the advocates of recognition great errors and misconceptions. It appears that these men are moved by the noblest of motives; they desire to permit the despised homœopathists to avail themselves of the superior knowledge and skill of the regulars when they have to treat a desperate case of illness; this very kind condescension of the time-honored regulars, who have found that their irregular brethren have made very decided inroads on their "*business*," is really and truly philanthropic—all merely for the benefit of the sick and the poor, helpless homœopaths. Will the regulars reflect for a moment? Do the regulars not know that the law of the land fully recognizes the new healing art promulgated by one of their own school, Samuel Hahnemann, and by him termed "*The Homœopathic Healing Art*," that it might appear from the outset that that school of medicine is governed by the law of the similars, and, so guided, has revolutionized the old therapeutics, which was governed by no law in particular save occasionally by the law of the contraries; that the graduate of the homœopathic colleges enjoys just the same rights, privileges, and immunities as are enjoyed by all graduates of medical schools? Do the regulars not perceive that not only their refusal to teach a progressive healing art in their medical schools, but their refusal to graduate medical students who had not only acquired all the knowledge taught by them in their schools, but who were furthermore found to have acquired additional knowledge of a new healing art, finally compelled the despised homœopaths to apply to a generous and just people for the charters of new colleges, medical schools, and hospitals, which were granted? And now the same men are found prohibiting the members of their organized medical societies to meet professionally or in consultation their equals before the law. These fossils set themselves up as a superior set of men and show by their persistent conduct that they are antagonistic to the will of the people, and, in reality, they violate every law when they inflict severe penalties on men of their school should they meet a homœopath professionally.

As long as the large majority of homœopathists honestly practiced their exclusive healing art, consultations with allopaths were *never* thought of, neither by the sick nor by the physicians—they were out of the question from obvious reasons, well understood. Then came a time when incompetent men, who saw the grand strides Homœopathy was making, if properly applied, adopted the system, but from indolence or incapacity to grasp the teachings of Hahnemann, many often failed sadly in their efforts to cure or relieve the sick. They then resorted to the palliative means used by the regulars, and while their betters through mistaken kindness failed to discipline them, they boldly attempted to declare Hahnemann's methods a failure, and that while the law of the similars might be a good guide at times, other means were to be used at the discretion of the physicians. The regulars were easily misled by these eclectics and were prone to believe that Homœopathy was disappearing as an exclusive healing art; and now we see the disinterestedness of the progressive regulars in making an effort to help to save the unfortunate sick who were so unlucky as to fall into the hands of men who professed to practice a healing art of which they knew nothing.

The public were sadly deceived by men who professed to be homœopaths, and who unhesitatingly resorted to non-homœopathic methods when their own shortcomings (and not the system of medicine they professed to practice) caused repeated failures. These men deceived the regulars and made them believe that Homœopathy, as an exclusive system of the healing art, was a failure; that true progress in medicine required the adoption of full freedom of medical opinion and action, and that the application of various, even antagonistic and conflicting, methods of therapeutics should be left to the individual judgment of the physician. Under this fallacious plea they asked for recognition by the regulars. The regulars looked upon this proposition in very different ways. To some it appeared in the light of a desire of the applicants gradually to return into the fold of allopathy, and that such a recognition was, if not paramount to the extermination of Homœopathy at once, at least as the first step in that direction; to others it appeared that the applicants had sailed under false colors and would likely continue to do so, and their past history did not recommend them as desirable companions. Let us leave these applicants for recognition to their fate, and let the regulars do with them as they think best.

We now speak for the homœopathicians who, like the early pioneers of our school, are living up to the methods taught by the master. These early pioneers and their followers gained recognition as healers and homœopathicians from the people because they *cured;* they *never* asked for any other recognition at the hands of the regulars than such as their superior successes demanded. However reluctantly such recognition has been given, we know of circumstances under which public opinion, based on observed, undeniable facts, has compelled them to utter such recognition. Repeated cures of cases given up or for a long time unsuccessfully treated by the regulars, come before an observing public, and they will wring a recognition out of them. *That* is the only *recognition* a homœopathician ever desires to effect, and this once fully established, the regulars will be compelled finally to recognize the homœopathic healing art as true, reliable, and exclusive. These desirable ends can only be obtained if we, as homœopathicians, are true to ourselves, and neither openly, nor even impliedly by silence, recognize any departures of the teachings of Hahnnemann, or deviate in the least from his inductive method. The community supposes that the homœopathic societies are the true exponents of that school of medicine. Erroneous ideas such as were accepted by the advocates of the new code can be obviated in the future if our own societies are true to the principles they promised to advocate when they were first established. If any society or any organized body, medical or national, allow their members to advocate new doctrines or laws in absolute contradiction to those on which the societies or organizations were established, if for reasons not explained these members are not called to account for their departures or heresies, the world at large will take it for granted that said societies or organizations indorse them. Let it be well understood that it is not necessary for a medical association to publicly indorse the departures and heresies of a member of said association by open resolutions. If a member of a medical association publishes and publicly proclaims false doctrines, departures, and heresies, he should be called to account for such acts by the association, and a failure to do so will be taken as an indorsement of such member and his false doctrines, departures, and heresies to the detriment of the association. Time will eventually clear up the *recognition muddle*, show whether or no the regulars in their charitable mood recognize the eclectic wing of the homœopaths, or whether the Institute will recognize the pathological picture-book advocates *vs.* our materia medica, or whether the I. H. A. will recognize the "potentizer advocates" *vs.* reliable pathogeneses, or whether all so-called homœopathic societies, colleges, and hospitals acknowledge Hahnemann's teachings as their sole and only guide for the further development of the healing art.

